# *Seladonia* (*Pachyceble*) *henanensis* sp. n. (Hymenoptera, Halictidae) from China

**DOI:** 10.3897/zookeys.305.4787

**Published:** 2013-05-28

**Authors:** Ryuki Murao, Osamu Tadauchi, Xu Huan-li

**Affiliations:** 1 Department of Biological Sciences, Faculty of Sciences, Kyushu University, Fukuoka, 812–8581 Japan; 2 Department of Entomology, College of Agronomy and Biotechnology, China Agricultural University, Beijing 100193, China

**Keywords:** Apoidea, Henan Province, taxonomy, new species, *Seladonia*

## Abstract

*Seladonia (Pachyceble) henanensis* **sp. n.**, is described from Henan Province, the eastern-central part of China. This species is separated from its allied species by a combination of the following morphological characters: head broad in female, inner hind tibial spur of female with 7–8 slender teeth, T1 basolaterally with appressed hair tuft in both sexes, and genitalia with long and large lower gonostylus in male. Important taxonomic characters are illustrated with photographs, scanning electron micrographs, and line drawings.

## Introduction

The halictine bee genus *Seladonia* Robertson (Halictidae, Halictinae) is a nearly cosmopolitan group, with 107 recognized species (Ascher and Pickering 2012). According to both morphological and molecularphylogenetic analyses (Pesenko 2004, Danforth et al. 1999, Gibbs et al. 2012), this genus is sister group to the genus *Halictus* Latreille. *Seladonia* differs from *Halictus* by the body having a metallic green or blue-green luster, posterior margin of fourth metasomal sternum straight, and male genitalia with medial lobe on upper gonostylus. In addition, *Seladonia* is divided into six subgenera (Pesenko, 2004): *Mucoreohalictus* Pesenko, 2004, *Pachyceble* Moure, 1940, *Paraseladonia* Pauly, 1997, *Placidohalictus* Pesenko, 2004, *Seladonia* s. str. Robertson, 1918, and *Vestitohalictus* Blüthgen, 1961. On the other hand, *Seladonia* is often regarded as a subgeneric rank of the genus *Halictus* (e.g., Michener 2007). Three subgenera (*Mucoreohalictus*, *Placidohalictus*, and *Vestitohalictus*) of the genus *Seladonia* sensu Pesenko (2004) corresponds to *Halictus (Vestitohalictus)* sensu Michener (2007), but the rest subgenera of it share with Michener’s classification (Michener 2007). We treat *Seladonia* at the generic level in this study, in accordance with Pesenko (2004).

Second and third authors, Tadauchi and Xu, visited Henan Province in the eastern-central part of China in 2011 for a collaborative project, and then they collected various wild bees in the area. First author, Murao had an opportunity to examine the specimens, one of which is a new *Seladonia* species belonging to the subgenus *Pachyceble*. In the present paper, we describe this new species and illustrate diagnostically important characters with drawings, photographs, and scanning electron micrographs.

## Material and methods

This study is based on the specimens deposited in the Entomological Laboratory, Faculty of Agriculture, Kyushu University, Fukuoka, Japan (ELKU), the late Dr. Shoichi F. Sakagami’s collection, deposited in the Museum of Nature and Human Activities, Sanda, Hyogo, Japan (MNHAH), and the first author’s private collection (without abbreviation). Terminology used in the description follows Sakagami and Ebmer (1987), and partly Pesenko (2006). Abbreviations used in the text are as follows:

BL = body length; WL = wing length; HL = head length; HW = head width; IOD = interocellar distance; OOD = ocellocular distance; OCD = ocelloccipital distance; UOD = upper interorbital distance; MOD = maximum interorbital distance; LOD = lower interorbital distance; CAL = clypealveolar distance; CPL = clypeal length; EL = eye length; EW = eye width; GW = genal width; Fn = nth antennal flagellomere; FnL = nth flagellomere length; FnW = nth flagellomere width; MsW = mesosomal width; SCL = mesoscutellar length; MNL = metanotal length; PDL = propodeal dorsum length; MtW = metasomal width; IS = interspace between punctures (IS 0.5 = means 1/2 of the diameter of punctures); PP= punctures. Body measurements are given in ranges followed by the average and standard deviation.

**Figures 1–6.**
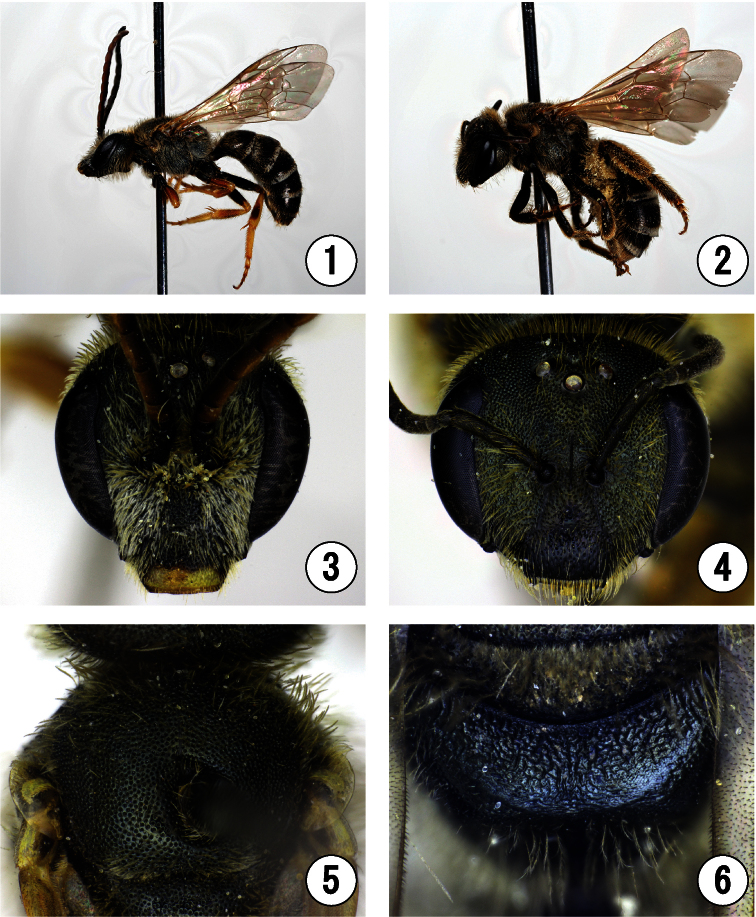
*Seladonia (Pachyceble) henanensis* sp. n. **1–2** lateral habitus **3–4** head in frontal view **5** mesoscutum **6** propodeal dorsum. **1, 3, 5** male, holotype. **2, 4, 6** female paratype.

Comparative material examined. *Seladonia (Pachyceble) tumulorum tumulorum* (Linnaeus, 1758). [SWITZERLAND] 2♀, Schweitz 450m Delémont Fangzeit, 23. vii. 1972 (E. Hüttinger, MNHAH). [AUSTRIA] 2♀, Umgeb. Linz, O. Öst., 31. vii. 1928 (H. Priesner, MNHAH); 1♀, Tirol, Innsbruck Weiherburg, 25. v. 1965 (A.W. Ebmer, MNHAH); 2♀, Oberöst., Linz-Koglerau, 8. vi. 1965 (A.W. Ebmer, MNHAH); 2♀1♂, Biologiezentrum, Linz, 48°20'13.86"N, 14°18'44.78"E, 8. vii. 2012 (R. Murao, 1♂ illustrated in Figs 19, 22); 1♀, Nickelsdorf-Kleylehof, Bulgenland, 12. vi. 1973 (A.W. Ebmer, MNHAH). [JUGOSLAVIA] 1♀, Istrien Ucka 1000m, 13. vii. 1971 (A.W. Ebmer, MNHAH).

*Seladonia (Pachyceble) tumulorum ferripennis* (Cockerell, 1929): [JAPAN] 7♀, Jozankei N. Sapporo, Hokkaido, 22. v. 1960 (M. Shiokawa, MNHAH), 19. vii. 1960 (M. Shiokawa, MNHAH), 3. vii. 1960 (M. Shiokawa, MNHAH), 31. vii. 1960 (M. Shiokawa, MNHAH); 3♀, University Campus, Sapporo, Hokkaido, 23. v. 1959 (S.F. Sakagami, MNHAH, paratype), 11. vi. 1959 (S. F. Sakagami, MNHAH), 20. viii. 1959 (S. F. Sakagami, MNHAH), 9. ix. 1959 (S.F. Sakagami, MNHAH).

*Seladonia (Pachyceble) leucahenea leucahenea* (Ebmer, 1972): [KAZAKHSTAN] 2♀, Aksu Valley, alt. 130–560m, S. Kazakhstan Prov., 16. vi. 2003 (O. Tadauchi, ELKU); 1♀, Karaalma, alt. 1210m, near Jabagly, S. Kazakhstan Prov., 17. vi. 2003 (O. Tadauchi, ELKU); 1♀, Achisai, alt. 670–700m, Mts. Karatau, S. Kazakhstan Prov., 4. vi. 2003 (O. Tadauchi, ELKU); 1♀, near Sternjak, Kokchetav District, N. Kazakhstan Prov., 19. vi. 2002 (V. Kazenas, A. Jdanko, and V. Rascheev, ELKU).

*Seladonia (Pachyceble) confusa confusa* (Smith, 1853): [U.S.A] 1♀, Lapeer Co., MICH., Deerfield twp., 30. v. 1937 (G. Steyskal, MNHAH); 1♂, IND. Hendricks Co. Pittsboro, 9. viii. 1964 (E. R. Jaycox, MNHAH, illustrated in Figs 20, 23).

*Seladonia (Pachyceble) confusa arapahonum* (Cockerell, 1906): [U.S.A] 2♀, Newton, Ut., 16. iv. 1948 (G. E. Bohart, MNHAH).

*Seladonia (Pachyceble) confusa alpina* (Alfken, 1907): [AUSTRIA] 1♀, Tirol, 4. v. 1965 (A. W. Ebmer, on *Salix* sp., MNHAH).

*Seladonia (Pachyceble) confusa perkinsi* (Blüthgen, 1926): [AUSTRIA] 5♀, Mt. Georgen/G, 9. iv. 1971 (A. W. Ebmer, MNHAH).

*Seladonia (Pachyceble) confusa pelagia* (Ebmer, 1996): [MONGOLIA] 5♀, Central aimak Songino, 24km SW von Ulan-Baator, alt. 1300m, 7. vi. 1966 (Z. Kaszab, ELKU).

## Taxonomy

### The subgenus Pachyceble

Moure, 1940

Pachyceble Moure, 1940: 54. Type species: *Pachyceble lanei* Moure, 1940, by original designation.

#### Diagnosis.

This subgenus is similar to the subgenus *Seladonia* s. str. in having the following characters: basitibial plate of female usually well developed; propodeal dorsum about as long as metanotum; propodeum usually not densely hairly from lateral to dorsal surfaces; metasomal terga usually with apical and basal bands of pale plumose hairs. But it is separated from them by male antenna long reaching metasoma, male F2 1.7–2.0 times as long as wide, and male S6 with deep depression behind gradulus.

### Seladonia (Pachyceble) henanensis

Murao sp. n.

urn:lsid:zoobank.org:act:11562BA6-82CD-4547-932D-DD01DF764DEB

http://species-id.net/wiki/Seladonia_henanensis

Figs 1 –18, 21

#### Type material.

Holotype: ♂, CHINA, Henan Prov., Xinxiang, Mt. Guanshan, Dongling Village, 35°33'34.709"N, 113°31'43.703"E, alt. 942m, 8. vii. 2011 (O. Tadauchi leg., ELKU: Code No. BeeFTadauchi01022, illustrated in Figs 1, 3, 5, 7, 9, 13–18, 21). Paratypes: [CHINA] 5♀1♂, same locality as the holotype, 9. vii. 2011 (O. Tadauchi, ELKU: Code Nos. BeeFTadauchi01015–01019 (♀), BeeFTadauchi01020 (♂); No. 01015 illustrated in Fig. 10, No. 01016 in Figs 11, 12, No. 01019 in Figs 2, 4, 6, No. 01020 in Fig. 8); 1♂, same locality as the holotype, 7. vii. 2011 (O. Tadauchi, ELKU: Code No. BeeFTadauchi01021).

#### Type depository.

The holotype and one female paratype (Code No. BeeFTadauchi01019) are deposited in the Department of Entomology, College of Agriculture and Biotechnology, China Agricultural University (Beijing, China), and the remaining paratypes are in ELKU.

#### Etymology.

The specific name is derived from the type locality, Henan Province of China.

#### Collecting sites.

All specimens were collected from along a sightseeing road at Guanshang National Geopark (Fig. 24).

#### Distribution.

China (Henan Province).

#### Diagnosis.

This species seems most closely related to the Trans-Palaearctic species *Seladonia (Pachyceble) tumulorum* and the Holarctic species *Seladonia (Pachyceble) confusa* in having similar body sculptures and male genitalia. However, it differs these allied species by the following key:

**Table:** 

1	♀♀	2
–	♂♂	4
2	T1 without appressed hair tuft	*Seladonia (Pachyceble) tumulorum*
–	T1 basolaterally with appressed hair tuft as in Fig. 10	3
3	HL/HW ratio= 0.93–1.00; inner hind tibial spur with 5–6 teeth	*Seladonia (Pachyceble) confusa*
–	HL/HW ratio= 0.88–0.90; inner hind tibial spur with 7–8 teeth	*Seladonia (Pachyceble) henanensis*
4	Male genitalia with short and small lower gonostylus (Fig. 22)	*Seladonia (Pachyceble) tumulorum*
–	Male genitalia with long or broad lower gonostylus (Figs 21, 23)	5
5	Lower gonostylus broad and truncate apically (Fig. 23)	*Seladonia (Pachyceble) confusa*
–	Lower gonostylus rounded apically (Fig. 21)	*Seladonia (Pachyceble) henanensis*

Based on the published works of Dawut and Tadauchi (2003), Ebmer (2005), Pesenko (2006), Pesenko and Wu (1997), and the specimens examined for this study, this species is also separated from the other Chinese species by the combination of following characters: HL/HW ratio= 0.88–0.90 in female; inner hind tibial spur of female with slender 7–8 teeth; upper gonostylus of male genitalia with slender medial lobe; and lower gonostylus of it long and large.

#### Description.

**Male. Coloration.** Head and mesosoma dark metallic green, metasoma black. Clypeus on lower half, and labrum yellow. Mandible outer surface dark yellow on basal 2/3, reddish brown apically. Scape and pedicel blackish brown; flagellomere dark yellowish brown on lower side, blackish brown on upper side. Tegula yellowish translucent. All coxae and trochanters black; fore and middle femora mostly yellow; hind femur mostly black except for apical 1/4; all tibiae and tarsi yellow. Wings transparent, veins and stigma pale yellowish. Metasomal terga narrowly yellowish brown translucent apically.

**Pilosity.** Body hairs whitish to pale yellowish. Head and mesosoma covered with elect fine branched hairs. Lateral surface of pronotum covered with thin tomentum. T1 basolaterally with dense, whitish appressed hairs, remaining areas with fine erect branched hairs. Disc of T2–T5 with sparse simple hairs. Apical bands on metasomal terga interrupted on all segments. Metasomal sterna without special hair tufts. Disc of S1–S4 with simple and short hairs which gradually dense toward the apical parts.

**Measurements** (n = 3, unit mm): BL = 6.43–8.29 (7.57±1.00), WL = 5.86–7.29 (6.38±0.79); HL = 2.10–2.25 (2.15±0.09), HW = 1.95–2.13 (2.04±0.09), IOD = 0.35–0.39 (0.37±0.02), OOD = 0.39–0.40 (0.39±0.01), OCD = 0.32–0.35 (0.33±0.02), UOD = 1.23–1.32 (1.27±0.05), MOD = 1.27–1.39 (1.33±0.06), LOD = 0.97–1.06 (1.02±0.05), CAL = 0.88–1.00 (0.95±0.07), CPL = 0.45–0.58 (0.51±0.06), EL = 1.35–1.45 (1.38±0.06), EW = 0.60–0.65 (0.63±0.03), GW = 0.30–0.45 (0.38±0.08), F1L = 0.19–0.21 (0.20±0.01), F2L = 0.32–0.34 (0.33±0.01), F3L = 0.34–0.37 (0.35±0.02), F10L = 0.34–0.35 (0.34±0.01), F2W = 0.19–0.21 (0.20±0.01); MsW = 2.05–2.25 (2.13±0.10), SCL = 0.50 (0.50±0.00), MNL = 0.30–0.33 (0.32±0.01), PDL = 0.35–0.40 (0.38±0.03); MtW = 1.85–2.15 (1.98±0.15).

**Structure.** Head nearly as long as wide; HW:HL = 1:1.05. Vertex rounded in frontal view. MOD:UOD:LOD = 1:0.96:0.77. IOD:OOD:OCD = 1:1.07:0.91. Ocellocular area and frons flat, dull, with reticulate PP. Paraocular area similar sculptures with ocellocular area and frons. Supraclypeus slightly convex with dense PP, IS smooth (IS = 0.2). CPL:CAL = 1:1.87. Clypeus gently elevated from middle to lower area, with dense PP, IS smooth (IS = 0.2–0.5). EW:GW = 1:0.61. Malar space short, 0.3 times as wide as mandible at base. Hypostoma distinctly striated. Mandible edentate. Labrum without basal elevation and distal process. Antenna long, reaching metasoma. F1–F3L:F10L:F2W = 1:1.70:1.81:1.75:1.04; flagellomere nearly flattened on lower side.

**Figures 7–12.**
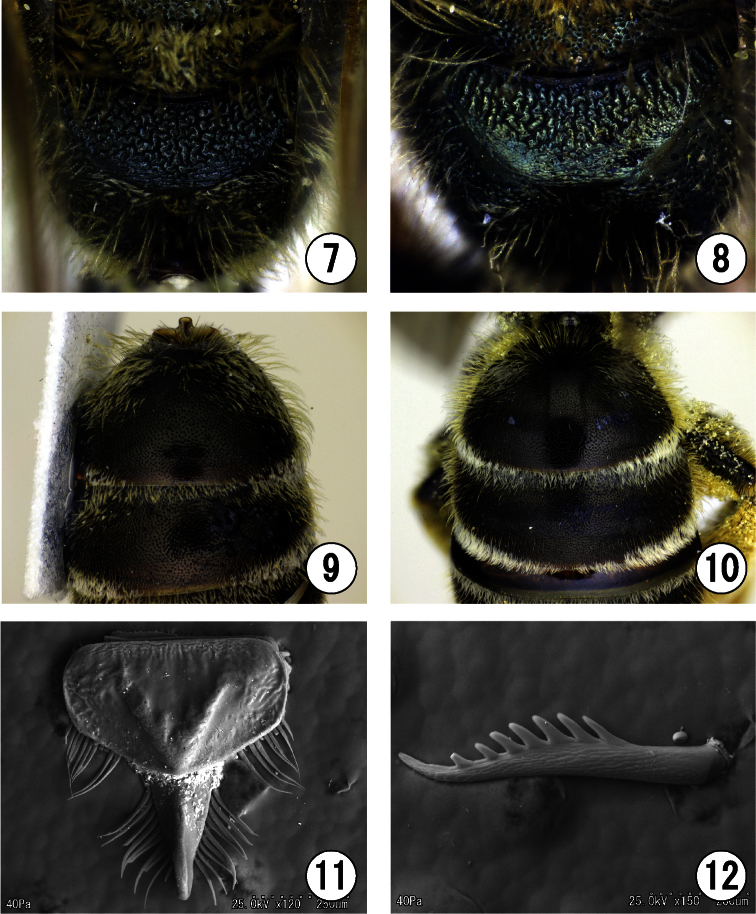
*Seladonia (Pachyceble) henanensis* sp. n. **7–8** propodeal dorsum **9–10** 1st and 2nd metasomal terga **11** labrum **12** inner hind tibial spur **7, 9** male, holotype **8** male, paratype **10–12** female, paratype.

Pronotal lateral ridge rounded, rather indistinct; lateral surface weakly rugulose below; lateral lobe apically rounded. Mesoscutum (Fig. 5) and mesoscutellum shiny, with dense PP over entire surface, IS smooth (IS = 0.2–0.8). Metanotum and mesepisternum dull and rugulose. SCL:MNL:PDL= 1:0.63:0.77. Propodeal dorsum (Fig. 7) slightly inclined, with distinct irregular sinuate ridges over entire surface. Propodeal side and shield densely punctured over entire surface, IS smooth (IS = 0.3–0.5). Hind tibia without basitibial plate. Inner hind tibial spur without distinct teeth. Hind tarsus slender.

**Figures 13–17.**
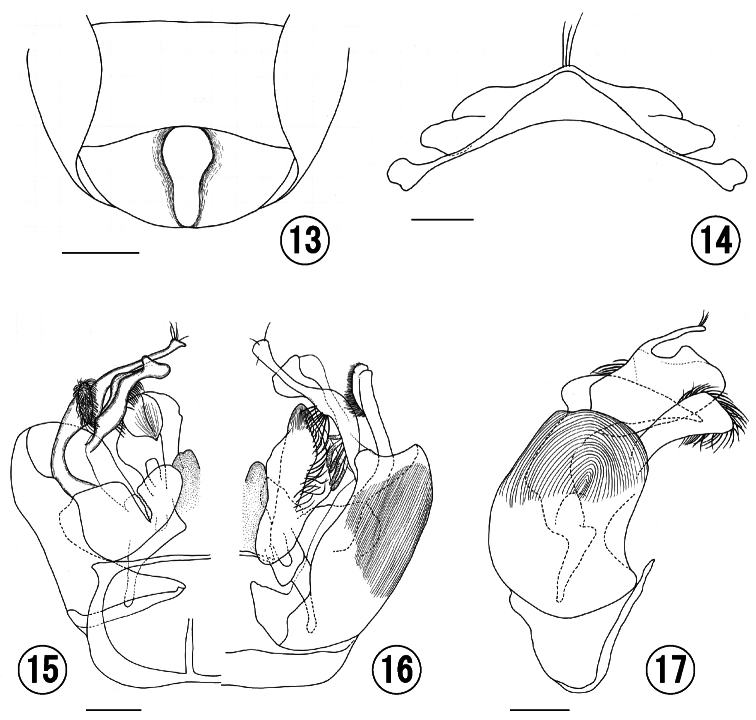
Male of *Seladonia (Pachyceble) henanensis* sp. n., holotype. **13** 6th metasomal sternum **14** 7th and 8th metasomal sterna **15** genitalia in ventral view **16** genitalia in dorsal view **17** genitalia in lateral view. Scale bars: **13**, 0.5 mm; **14–17**, 0.2 mm.

Discs of T1–T3 with dense PP over entire surface, IS smooth (IS = 0.5–1.0). S1–S4 distinctly tessellate over entire surface. S1–S4 apically nearly straight, S5 increasingly incurved. S6 (Fig. 13) with a distinct longitudinal median depression. S7–S8 (Fig. 14): S7 medially triangular, apex not exceeding S8; S8 medially slightly projecting, apex rounded with a few hairs as long as S8 itself.

**Figures 18–24.**
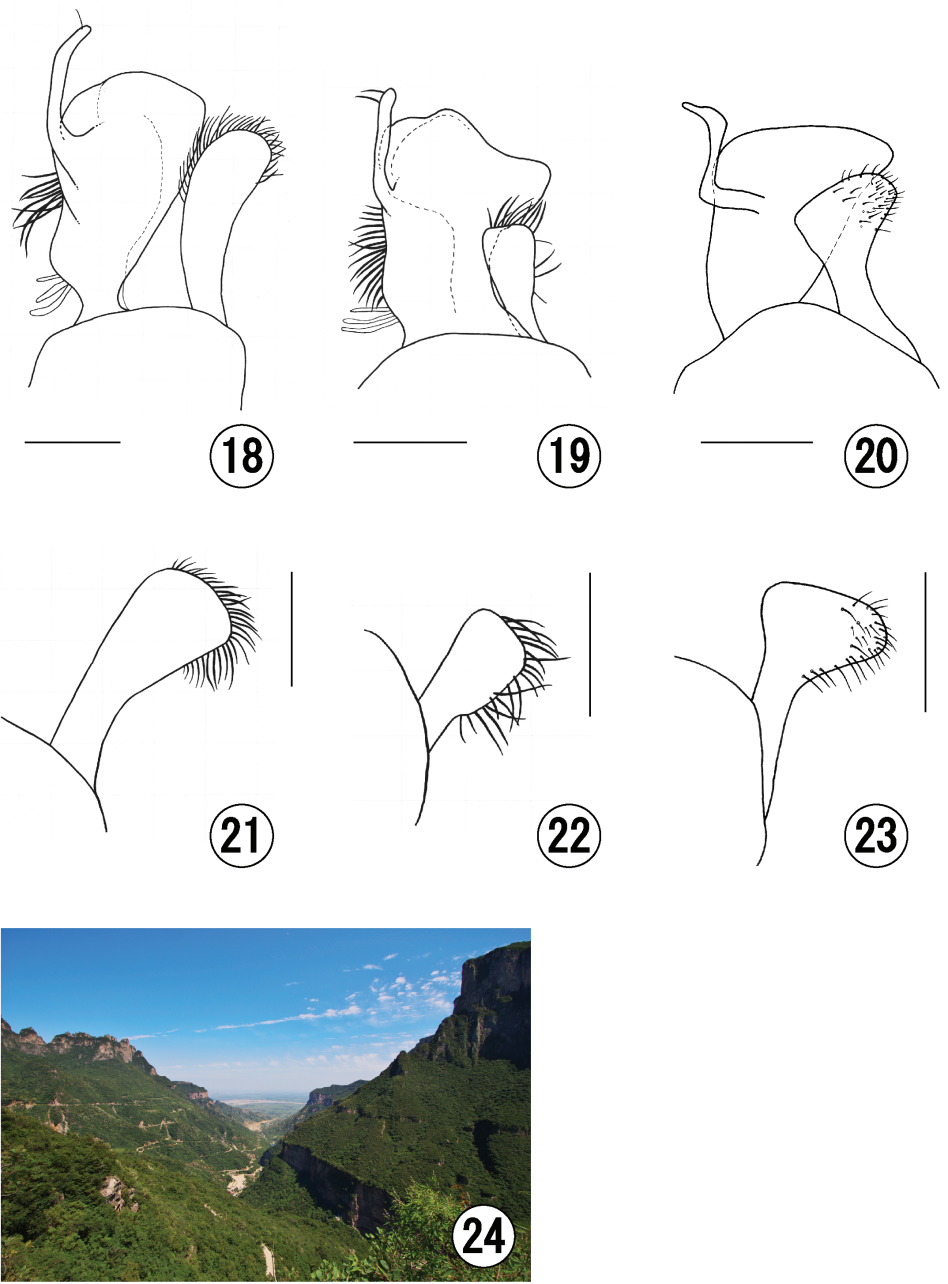
**18, 21** Male of *Seladonia (Pachyceble) henanensis* sp. n., holotype **19, 22** Male of *Seladonia (Pachyceble) tumulorum tumulorum* (Linnaeus) **20, 23** Male of *Seladonia (Pachyceble) confusa confusa* (Smith) **24** Collecting site of *Seladonia (Pachyceble) henanensis* sp. n., photograph by Dr. Satoshi Kamitani **18–20** gonostylus of male genitalia in dorsal view **21–23** lower gonostylus of male genitalia in lateral view. Scale bars: 0.2 mm.

**Genitalia** (Figs 15–18, 21): gonobase short, ventral arm connected with each other in upper ends; gonocoxite nearly parallel-sided on inner and outer margins in dorsal view, with longitudinal striation in dorsal view, with fingerprint striation in lateral view; upper gonostylus with dense tuft of short hairs and another tuft of several long filament-like modified hairs ventrally, medial lobe slender with a few short hairs apically; lower gonostylus 2.3 times as long as wide, nearly as high as upper gonostylus, spatulate in lateral view, and with dense short hairs on inner surface; penis valve broad medially, with relatively long hairs along median line in dorsal view.

**Female.** As in male except as indicated.

**Coloration.** Clypeus and all legs without yellow maculatons. Mandible dark reddish, remainder black. All segments of antenna dark brown.

**Pilosity.** Scopa well developed on hind femur to tibia, and S1–S3. Apical bands on metasomal terga interrupted on T1–T2, completed but narrow on T3–T4.

**Measurements** (n = 5): BL = 7.71–9.29 (8.60±0.60), WL = 6.43–7.86 (7.37±0.60); HL = 2.15–2.30 (2.25±0.06), HW = 2.40–2.60 (2.53±0.08), IOD = 0.35–0.42 (0.41±0.03), OOD = 0.45–0.52 (0.49±0.03), OCD = 0.35–0.42 (0.41±0.03), UOD = 1.52–1.65 (1.57±0.05), MOD = 1.66–1.81 (1.75±0.06), LOD = 1.42–1.55 (1.48±0.06), CAL = 0.85–0.90 (0.88±0.03), CPL = 0.45–0.50 (0.47±0.02), EL = 1.50–1.55 (1.53±0.03), EW = 0.65–0.75 (0.71±0.04), GW = 0.45–0.65 (0.54±0.08), MsW = 2.40–2.60 (2.53±0.08), SCL = 0.50–0.55 (0.54±0.02), MNL = 0.30–0.35 (0.34±0.02), PDL = 0.35–0.40 (0.39±0.02); MtW = 2.50–2.85 (2.75±0.15).

**Structure.** Head wider than long; HW:HL = 1:0.89. Vertex flat medially in frontal view. MOD:UOD:LOD = 1:0.90:0.85. IOD:OOD:OCD = 1:1.21:1.00. PP on supraclypeal area gradually becoming sparse to apically, IS = 0.5–1.0. CPL:CAL = 1:1.89. Clypeus nearly flat, its punctures sparser than in male, IS = 0.5–2.0. EW:GW = 1:0.76. Malar space linear. Hypostoma distinctly tessellate. Mandible bidentate. Labrum (Fig. 11): basal area of labrum 1.7 times as wide as long; basal elevation developed, triangle-shaped; distal process slender, slightly shorter than basal area (0.9 times), and without lateral projection; keel of distal process obtuse apically; labral fimbria acutely pointed at apex. Antenna short, its apically not reaching mesoscutellum as well as the other congeners.

Lateral surface of pronotum finely punctuated, IS smooth. SCL:MNL:PDL = 1:0.62:0.72. Propodeal dorsum (Fig. 6) without smooth area. Propodeal side with dense PP on lateral slope, with weak rugulae on rest parts. Propodeal shield weakly tessellate, with sparse PP. Inner hind tibial spur (Fig. 12) with 7–8 slender teeth.

Luster on T3–T4 duller than T1–T2. PP on T1 sparser than T2–T4; IS = 1–2.5 on T1, IS= 0.5 on T2–T4. Metasomal sterna flat, weakly tessellated over entire surface, and nearly straight apically.

**Variation.** Male hypostoma striate in holotype, but nearly smooth in one paratype. In addition, male propodeal dorsum without smooth area (Fig. 7) in holotype, but with narrow and smooth area along posterior margin (Fig. 8) in two paratypes.

##### List of Chinese species of *Seladonia (Pachyceble)*

*1.* *Seladonia (Pachyceble) argilos* (Ebmer, 2005)

*2.* *Seladonia (Pachyceble) confusa pelagia* (Ebmer, 1996)

*3.* *Seladonia (Pachyceble) henanensis* Murao, sp. n.

*4.* *Seladonia (Pachyceble) leucahenea leucahenea* (Ebmer, 1972)

*5.* *Seladonia (Pachyceble) opacoviridis* (Ebmer, 2005)

*6.* *Seladonia (Pachyceble) tibetana* (Blüthgen, 1926)

*7.* *Seladonia (Pachyceble) tumulorum ferripennis* (Cockerell, 1929)

*8.* *Seladonia (Pachyceble) yunnanica* (Pesenko & Wu, 1997)

*Seladonia (Pachyceble) confusa alpina* (Alfken, 1907) and *Seladonia (Pachyceble) dorni* (Ebmer, 1982) are recorded from China (Fan 1991, Niu et al. 2004). However, these records not include in above list, in accordance with Ebmer (2005) and Pesenko (2006).

## Supplementary Material

XML Treatment for Pachyceble

XML Treatment for Seladonia (Pachyceble) henanensis
